# A Simple Method for the Acquisition and Transmission of Brassica Yellows Virus from Transgenic Plants and Frozen Infected Leaves by Aphids

**DOI:** 10.3390/plants10091944

**Published:** 2021-09-18

**Authors:** Deng-Pan Zuo, Meng-Jun He, Xiang-Ru Chen, Ru-Jian Hu, Tian-Yu Zhao, Xiao-Yan Zhang, Yan-Mei Peng, Ying Wang, Da-Wei Li, Jia-Lin Yu, Cheng-Gui Han

**Affiliations:** 1 State Key Laboratory for Agrobiotechnology, Ministry of Agriculture and Rural Affairs Key Laboratory of Pest Monitoring and Green Management, College of Plant Protection, China Agricultural University, Beijing 100193, China; B20173190806@cau.edu.cn (D.-P.Z.); 18864805798@163.com (M.-J.H.); chxr8628@163.com (X.-R.C.); hurujian@cau.edu.cn (R.-J.H.); ty_zhao@163.com (T.-Y.Z.); xiaoyan433@163.com (X.-Y.Z.); pengyanmei7628@163.com (Y.-M.P.); yingwang@cau.edu.cn (Y.W.); 2 State Key Laboratory of Agrobiotechnology, Ministry of Agriculture Key Laboratory of Soil Microbiology, College of Biological Sciences, China Agricultural University, Beijing 100193, China; Dawei.Li@cau.edu.cn (D.-W.L.); yjl@cau.edu.cn (J.-L.Y.)

**Keywords:** Brassica yellows virus, *Myzus persicae*, transgenic plants, frozen BrYV-infected plants, acquisition and transmission

## Abstract

Brassica yellows virus (BrYV) is a tentative species of the genus *Polerovirus*, which occurs widely, and mostly damages *Brassicaceae* plants in East Asia. Because BrYV cannot be transmitted mechanically, an insect-based transmission method is required for further virus research. Here, a reliable and unrestricted method is described, in which non-viruliferous aphids (*Myzus persicae*) acquired BrYV from transgenic *Arabidopsis thaliana*, harboring the full-length viral genome germinated from seeds and its frozen leaves. The aphids then transmitted the virus to healthy plants. There was no significant difference in acquisition rates between fresh and frozen infected leaves, although the transmission rate from frozen infected leaves was lower compared to fresh infected leaves. This simple novel method may be used to preserve viral inocula, evaluate host varietal resistance to BrYV, and investigate interactions among BrYV, aphids, and hosts.

## 1. Introduction

Viruses belonging to the *Polerovirus* genus (family *Luteoviridae*) cause plant diseases that are notoriously difficult to manage [1]. Poleroviruses are positive single-stranded RNA viruses. The virions are spherical, approximately 25–35 nm in diameter, and non-enveloped. The virions are composed of a coat protein and genomic RNA (gRNA) [2]. Poleroviruses are distributed worldwide and can infect a variety of plants, such as those of Cruciferae, Polygonaceae, Cucurbitaceae, and Gramineae families, and cause serious diseases and economic losses [3,4,5]. Poleroviruses cannot be transmitted mechanically under natural conditions. They are only transmitted by aphids in a circulative, non-propagative manner. However, they may be spread by agroinfiltration or grafting under artificial conditions [6,7].

Brassica yellows virus (BrYV) is a tentative newly identified species in the genus of *Polerovirus*, and it is closely related to turnip yellows virus (TuYV) [8,9]. It is spread widely throughout China as well as in South Korea and Japan [4,10,11]. BrYV has a wide host range, although it mainly infects *Brassicaceae* crops, including cabbage (*Brassica oleracea* var. *capitata*), Chinese cabbage (*B. pekinensis*), cauliflower (*B. oleracea* var. *botrytis*), mustard (*B. juncea*), and turnip (*Raphanus sativus* var. *oleifera*), usually causing leaf malformations and yellowing in the field [4,9,10,11,12]. BrYV has at least three genotypes (BrYV-A, B, and C) as determined by sequence analyses, and full-length infectious cDNA clones of these three genotypes have been developed successfully [8,10,11,13,14]. The full-length amplicon of BrYV-C has been successfully transferred into *Arabidopsis thaliana*, resulting in two stable transgenic lines, 111 and 412, which exhibit severe symptoms, including dwarfism and purple leaves [15]. The BrYV-encoded P0 protein interacts with plant S-phase kinase associated protein 1, contributing to its stability, which allows it to evade autophagy and proteasomal degradation [16]. In addition, the P0 protein impairs the antiviral activity of *Nicotiana benthamiana* rubisco assembly factor 2 by altering its localization pattern to facilitate viral infection [17].

Poleroviruses are phloem-limited and produce low titers in infected plants; therefore, the storage and utilization of the viral inocula sources are important [6]. Moreover, field-collected infected plants are not usually infected by a single virus, and this greatly limits the simplicity and reliability of research on poleroviruses. Van den Heuvel et al. (1991) reported the transmission and acquisition of potato leafroll virus (PLRV) by *Myzus persicae* from infected *Parietaria floridana* and artificial diets containing the purified virus [18]. Similarly, Boissinot et al. (2017) reported the extraction of virions from infected plants or *Agrobacterium tumefaciens* bacteria containing infectious viral clones to infect plants to feed non-viruliferous aphids [19]. *Agrobacterium tumefaciens*-mediated transformation of full-length cDNA copy of the genome of PLRV was introduced into the genome of potato and tobacco plants, resulting in viral titers that were higher than the non-transgenic (WT) PLRV-infected plants. Moreover, the virus in the transgenic plants can spread to the stem epidermal cells and readily transmitted PLRV through aphid feeding to tested plants [20]. Shikata et al. (1977) reported that the rice black-streaked dwarf virus (RBSDV) isolated from infected rice was investigated after being injected into the small brown planthopper (SBPH), which could transmit the virus to healthy plants [21]. Moreover, the SBPH directly acquired RBSDV from frozen infected rice leaves and transmitted the virus to a susceptible rice variety [22]. Zhang et al. (2007) provided a simple, rapid, and reliable method by which virus-free SBPH acquired the rice stripe virus from frozen infected rice leaves and transmitted the virus to healthy rice plants [23]. These reports provided valid methods to address the problem of viral acquisition by insect vectors to establish basic methods for breeding and genetic research on plant disease resistance. Until now, there have been no reports that aphids acquire BrYV from transgenic plants harboring the full-length viral genome or from their frozen infected leaves. BrYV infectious cDNA clones infect model plants, like *A. thaliana* and *Nicotiana benthamiana* through *A. tumefaciens*-mediated infiltration [14,15]. However, the *A. tumefaciens* infiltration method cannot completely simulate natural viral inoculation conditions. Therefore, we developed a simple and reliable method for the acquisition and transmission of BrYV by aphids from transgenic and frozen infected plants. This method will assist in BrYV inocula preservation, transmission and allow the study of interactions among the virus, aphids and hosts. In addition, this method provided a foundation for the establishment of similar transmission systems for other poleroviruses.

## 2. Results

### 2.1. Viral Inocula Were Ready for Transmission

Two BrYV inocula were selected for aphid feeding and transmission. First, the seedlings of the seventh generation transgenic *A. thaliana* line 412 harboring the BrYV full-length cDNA clone under control of the ubiquitous 35S promoter and showing typical purple symptoms, were used as fresh BrYV inocula [15]. Then, the transgenic plants were kept at −20 °C as frozen BrYV inocula. Next, the full-length infectious BrYV clone was transferred to *A. tumefaciens* C58CI and inoculated into *A. thaliana*. At 14 days after inoculation, RT-PCR was used to detect BrYV in the inoculated and systemic leaves. The infection incidence in *A. thaliana* inoculated leaves by BrYV was 86.34%, while the incidence in systemic leaves was only 46.75% (Table 1). Therefore, transgenic *A. thaliana* line 412 and an *A. tumefaciens*-mediated BrYV infectious clone can be used as inoculation materials for aphid feeding and viral transmission.

### 2.2. The Aphid Species Was Confirmed and a Non-Viruliferous Aphid Clonal Population Was Obtained

The aphids containing BrYV were identified as green peach aphids (*M. persicae*), and a non-viruliferous aphid clonal population Mp433-1 was obtained. DNA was extracted from a single aphid and universal primers (LEP-F and LEP-R) for the mitochondrial cytochrome oxidase gene (*COI*) were used for PCR amplification. The sequencing results were analyzed using the BLAST algorithm in NCBI, and the nucleotide sequence shared 99% identity with *M. persicae* strain YL. The cabbage *COI* (accession number: KM577343) indicated that the aphid species was green peach aphid (*M. persicae*). The aphid colony was screened to obtain virus-free and clonal aphids. A single aphid was identified for ovipositing and the progeny were subsequently reared on healthy turnip plants. Then, one nymph was transferred to healthy *A. thaliana*, and for approximately 5–10 generations, aphids were randomly selected and analyzed by RT-PCR to confirm the absence of BrYV. For another 5–10 generations, the offspring of these non-viruliferous aphids were classified as a clonal population, named *M. persicae* isolate Mp433-1. The selected aphid offspring from the single aphid were confirmed to be BrYV negative by RT-PCR with BrYV-specific primers (BrY4964F/BrY5635R).

### 2.3. Acquisition and Transmission of BrYV by the Aphids Were Available

Aphids were fed on the frozen leaves of the transgenic *A. thaliana* line 412 preserved for 180 d and 270 d, fresh leaves of line 412 and leaves of healthy *A. thaliana* for 2 days (60 aphids per treatment) and each experiment was repeated for three times. The mean numbers of living aphids were 35.33, 30.33, 55.33, and 51.33, respectively, and survival rates of 58.59%, 50.56%, 92.22%, and 85.56%, respectively (Table 2). Subsequently, 16 aphids from each treatment were selected randomly to independently inoculate 3–4-week-old healthy *A. thaliana*. At 2 days after inoculation, the presence of BrYV in the aphids was confirmed by RT-PCR. The mean survival rates of viruliferous aphids fed on line 412 and 180-d and 270-d frozen infected leaves were 93.75%, 87.5%, and 77.08%, respectively. Each experiment was performed three times (Table 2). There were no significant differences among the three treatments. Thus, both the fresh and frozen 412 leaves may be used for aphid BrYV acquisition, although the frozen leaves affected the aphid survival rate compared with the fresh leaves under the same experimental conditions.

**Figure 1:**
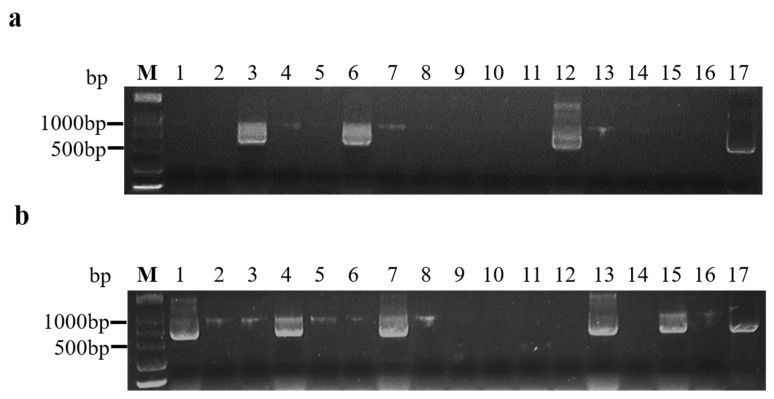
Agarose gel analysis of RT-PCR products using BrYV primers from *A. thaliana* plants inoculated by aphids. (**a**) Aphids that acquired the virus from frozen infected leaves; (**b**) aphids that acquired the virus from transgenic *A. thaliana* line 412 leaves. M: Marker (DCL 2000, Tsingke, Beijing, China); lines 1–16: aphids inoculated through *A. thaliana*; line 17: positive control.

At 14 days after inoculation, 16 inoculated *A. thaliana* were tested for BrYV infections using RT-PCR. A product of the expected size was amplified from infected plants but not from non-symptomatic plants (Figure 1a,b). The transmission rates to plants by aphids fed on line 412 and 180-d and 270-d frozen infected leaves were 33.33%, 16.67%, and 10.42%, respectively (Table 2). Thus, the aphids acquiring the virus from the BrYV-transgenic and frozen leaves transmitted the virus to healthy plants, producing symptoms that were identical to those of *A. tumefaciens*-mediated BrYV-infected and BrYV-transgenic plants.

### 2.4. The Greatest Transmission Efficiency of BrYV Was Determined by Assessing Minimal Aphid Numbers and Inoculation Times

**Figure 2:**
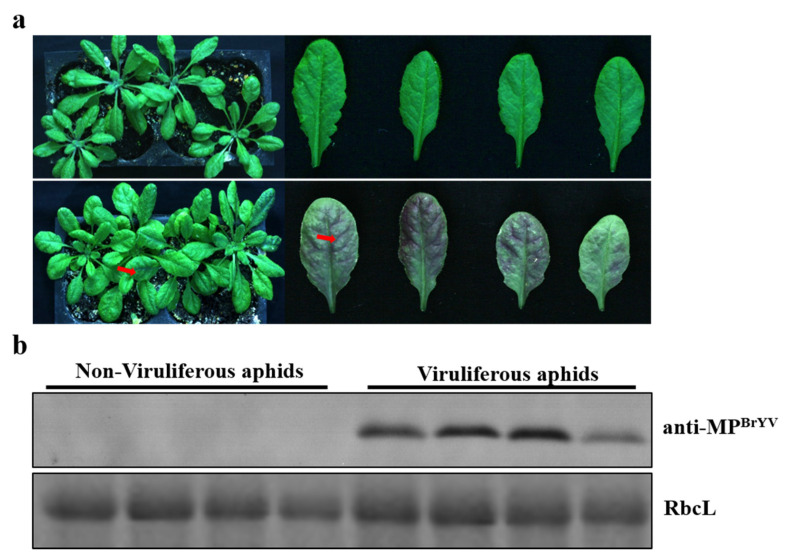
Symptoms and virus detection in *A. thaliana* inoculated with BrYV by aphids. (**a**) Typical symptoms of purple leaves on *A. thaliana* plants at 14 days post-inoculation by six viruliferous aphids. (**b**) Western blotting analyses of the accumulation of BrYV MP extracted from *A. thaliana* upper leaves.

To ensure the greatest transmission efficiency, inoculations with different numbers of viruliferous aphids were performed. The aphids fed on fresh 412 plants for 2 days, and then, 1, 2, 4, 6, and 10 aphids (second–third instar) were transferred independently to *A. thaliana* to act as inoculants. At 2 days after inoculation, the aphids were eliminated using an insecticide. At 14 days after inoculation, RT-PCR showed that the rates of infected *A. thaliana* produced by 1, 2, 4, 6, and 10 aphids were 30%, 80%, 90%, 100%, and 100%, respectively (Table 3). The experiments were repeated three times per group. The presence of six viruliferous aphids resulted in an infection efficiency of 100%. Typical symptoms were purple leaves on *A. thaliana* plants inoculated with six viruliferous aphids by 14 days post-inoculation, and western blotting revealed that the virus transmitted by aphids successfully infected *A. thaliana* (Figure 2a,b). The tested minimal inoculation times required for six viruliferous aphids to transmit the virus, to healthy *A. thaliana* plants were 6, 12, 24, and 48 h. At 14 days after inoculation, the RT-PCR results showed that the transmission rate for the six aphids with inoculation times of 6, 12, 24, and 48 h were 12.5%, 50%, 62.5%, and 100%, respectively (Table 4), indicating that six viruliferous aphids present for 2 days resulted in a 100% BrYV-infection rate for *A. thaliana* plants.

As previous report, BrYV could be detected in Mustard (*Brassica juncea* var. *tumida*) and Chinese cabbage (*Brassica campestris* L. ssp. *Chinensis*) [4,13]. To test whether aphids fed on fresh 412 could transmit BrYV to its natural host plants, six aphids fed on the transgenic *A. thaliana* line 412 were transferred to mustard and Chinese cabbage. At 3 days after inoculation, aphids were eliminated using an insecticide. At 14 days after inoculation, RT-PCR showed that the rates of infected Mustard and Chinese cabbage were 94.4% and 100% respectively (Table 5). The experiment was repeated three times per group, indicating that viruliferous aphids could successfully transmit BrYV to its natural host plants.

## 3. Discussion

For plant virology research, virus preservation and utilization are very important [24,25]. Plant viruses in the *Polerovirus* (family *Luteoviridae*) cause emerging diseases that have serious economic consequences for many staple, vegetable, ornamental, and fruit crops, and the transmission by aphids is classified as persistent, circulative, and non-propagative [1,5,6]. Convenient tests to determine viral acquisition and transmission have not been available owing to the lack of viral inocula preservation. Therefore, it is important to explore a simple method for *Polerovirus* preservation and transmission. The technique presented here easily allows virus preservation and transmission by using transgenic plants and frozen infected leaves harboring the viral genome. The purpose of preservation is not only to maintain the infectivity of the virus, but also to eliminate mutations and contamination.

In this research, a very simple and feasible method was established using transgenic *A. thaliana* harboring the full-length BrYV genome and frozen infected leaves for aphid feeding and transmission. Previous research by Franco-Lara showed that PLRV genomic RNA transfers into common tobacco and potato, in which the viral genomic RNA and proteins undergo replication and translation. Furthermore, aphids (*M. persicae*) fed on the transgenic tobacco plants readily transmit PLRV to test plants, and the inoculation efficiency of five aphids present for 3 days was approximately 71% [20]. Boissinot extracted the virions from TuYV-infected plants, fed them to aphids, and then used 10 *M. persicae* (Sulzer) for 4 days for inoculations [19]. However, the method is technically demanding and requires viral purification equipment and instruments. In this study, six aphids (*M. persicae*) feeding in BrYV-transgenic *A. thaliana* for 2 days resulted in a transmission rate of up to 100% (Table 3), indicating that aphids can readily acquire the virus from transgenic *A. thaliana* plants. More conveniently, BrYV-transgenic plants may be germinated from transgenic *A. thaliana* seeds at any time for aphid feeding. The survival rate of aphids fed frozen diseased leaves was 58.89%, which was lower than that of aphids fed on the fresh infected leaves and fresh healthy leaves. Although the materials subjected to freezing conditions may affect aphid survival, there were no significant differences in the aphid viral acquisition rates (Table 2). The greatest survival rate for *M. persicae* occurred after feeding on detached fresh infected leaves, perhaps because detached leaves infected with the virus had a lower water-loss rate. However, transmission rates for aphids fed on the frozen infected leaves were lower than those fed on the fresh infected leaves, indicating that viral acquisition form the frozen samples was difficult (Table 2). Zhou’s previous work showed no significant difference in viral transmission between the SBPH fed on frozen or fresh infected rice leaves. The infected rice leaves had been preserved at −70 °C for 45 and 140 days [22]. Shikata et al. (1977) also showed that SBPH acquires viruses from 232-day frozen leaf tissues of RBSDV-infected rice leaves [21]. In this research, a single aphid acquired the virus from fresh infected leaves and 270-day frozen infected leaves, revealing a simple and feasible method for viral acquisition by aphids at any time, and the infected plants could be preserved at −20 °C for at least 270 days. This work may be further improved by feeding aphids on BrYV-infected leaves stored at −70 °C. *Agrobacterium tumefaciens*-mediated viral inoculation of *A. thaliana* and virus genomic transgenic plants may serve as a viral source for aphids that could successfully transmit it to its natural hosts. Because Polerovirus-infected plants are very difficult to preserve, this method overcomes the technical bottlenecks of viral transmission and preservation, and it can be used for screening resistant host varieties and for genetic analyses of a variety’s resistance to BrYV.

This method provides a simple and reliable approach using transgenic *Arabidopsis* and frozen leaves harboring the full-length BrYV genome for viral acquisition and transmission by aphids. This novel method can be applied to the preservation of viral inocula, evaluation of host variety resistance, and biological research on interactions among BrYV, aphids, and hosts. It may also provide a foundation for establishing similar methods for research on other poleroviruses.

## 4. Materials and Methods

### 4.1. Viral Resources

A 35S promoter-derived expression cassette containing the full-length cDNA amplicon of BrYV was transformed into *Arabidopsis* using the floral dip method as previously described and the seventh generation of line 412 seeds that constitutively expressed the viral genomic RNA of BrYV were used [12,14]. The 3–to 4-week-old *A. thaliana* line 412 seedlings grown in a greenhouse or a chamber were used as viral inocula. Alternatively, 4- to 5-week-old *A. thaliana* plants infiltrated with *A. tumefaciens* strain C58CI containing the full-length BrYV cDNA clone were used as viral inocula. All plants were grown under a 24 °C/14-h light and 22 °C/10-h dark cycle.

### 4.2. Identification of Aphid Species

In October 2017, aphids were collected from the west campus of China Agricultural University and maintained on turnip variety ‘Yamei No. 1’. For aphid species identification, we used PCR to amplify aphid *COI* genes. First, total DNA of a single aphid was homogenized in CTAB DNA extraction buffer, and aphid genomic DNA was isolated using a CTAB method [26]. Then, 300 μL of CTAB DNA extraction buffer was placed in the tube and mixed well by shaking on a 65 °C heater for 30 min. It was then mixed by turning the tube upside down several times. An equal volume of Tris-phenol was added, and the tube was centrifuged at 12,000× *g* for 15 min. Then, 300 μL supernatant was aspirated. Afterward, an equal volume of isopropanol was added and left at room temperature for 10 min. After centrifugation, the pellet was washed sequentially with 75% and 100% ethanol. Finally, the DNA was dried at room temperature, and then, 30 μL ddH_2_O was added to dissolve the pellet. A mitochondrial universal primer pair (LEP-F: ATTCAACCAATCATAAAGATATTG; LEP-R: AAACTTCTGGATGTCCAAAAAATCA) was used to amplified the aphid *COI* by PCR. PCR was carried out in a 25-μL mixture containing 12.5 μL 2× TSINGKE Master Mix (blue) (Tsingke Biological Technology Company, Beijing, China). PCR products were removed from agarose gels using a Gel Extraction Kit (OMEGA, Georgia, USA) and sent for sequencing. The sequences were then analyzed using the BLAST algorithm at NCBI (https://www.ncbi.nlm.nih.gov/) on 18 January 2018.

### 4.3. Generation of the Non-Viruliferous Aphid Clonal Population

An aphid colony was separated into virus-free and monoclonal aphids as described previously [18,27]. Here, we selected one single aphid from the aphids identified as *M. persicae* for oviposition, and its progeny were reared on healthy *A. thaliana*. Then, one nymph was transferred to healthy *A. thaliana*, and for approximately 5–10 generations, aphids were randomly selected and analyzed by RT-PCR to confirm the absence of BrYV. Afterward, one non-viruliferous aphid was reared on healthy turnip seedlings (3–4-leaf stage) in a light incubator at 24 °C with a 16-h light/8-h dark cycle. For approximately 10 offspring generations, the non-viruliferous aphids formed the population named *Mp433-1*. The selected aphid offspring were also confirmed to be negative for BrYV by RT-PCR. Total RNA was extracted from the aphid samples using TRIzol reagent in accordance with the manufacturer’s instructions (Invitrogen). First, a single aphid was placed into a 2.0 mL Eppendorf tube and a steel ball was added. Then, the sample was placed in liquid nitrogen, ground, and immediately centrifuged at 12,000 rpm in a precooled centrifuge. After a 10-s centrifugation, 300 μL TRIzol was added to the sample, and the sample was allowed to be fully lysed. Then, 200 μL of chloroform was added and shaken for 30 s. The sample was placed at room temperature (25 °C) for 5 min and then centrifuged at 4 °C for 10 min. Afterwards, the supernatant was removed, placed in a new RNase-free tube and 300 μL of isopropanol was added. The sample was placed at room temperature for 10 min and then centrifuged at 4 °C for 15 min. The supernatant was discarded and 1 mL 75% alcohol was added to lightly wash the RNA pellet before centrifuging at 12,000× *g* for 5 min. Finally, the supernatant was removed, and the pellet was dried at room temperature for 5–10 min. It was then dissolved in 20 μL of DEPC-pretreated water. The reverse transcription system was used in accordance with the M-MLV reverse transcriptase’s instructions (Promega). PCR detection with specific primers (BrY4964F: CATAAAGCCCTTGTCGGCGAG, BrY5635R: GTAGAACACTCGTTGCCTATCC) was performed to detect the transcriptional products.

### 4.4. Viral Acquisition Experiments

The non-viruliferous aphid group (*Mp433-1*) was confirmed by RT-PCR before feeding. The BrYV-transgenic *A. thaliana* grown for 3–4 weeks were used, and detached leaves were placed in plantlet bottles for aphid feeding. Frozen infected leaves (frozen for 180 and 270 days) were thawed in Petri dishes containing wet filter paper, which retained moisture, for at least 4 h and were then transferred to plantlet bottles in a light incubator. In total, 60 non-viruliferous aphids (second–third instar) were placed into plantlet bottles. The blank controls were fresh healthy plant leaves. After a 48-h acquisition-feeding period, the numbers of surviving aphids were calculated, and the aphids were transferred from the inocula leaves to healthy *A. thaliana* seedlings using a brush. Each experiment was repeated at least three times.

### 4.5. Viral Transmission Experiments

After feeding individually on line 412, as well as frozen and fresh infected *A. thaliana* leaves, aphids were caged for virus detection. To determine the highest BrYV transmission efficiency, wild type *A. thaliana* plants were inoculated independently using 1, 2, 4, 6, and 10 aphids (second–third instar). After inoculation, aphids were eliminated using an insecticide. Each seedling was inoculated individually for 2 days. After symptom investigation, the presence of the virus in aphid-exposed plants was detected using RT-PCR (Figure 1a,b). To determine if aphids could transmit BrYV to its host plants, Mustard and Chinese cabbage (Cultivated variety) were inoculated using six aphids. The aphids that fed on healthy leaves were used as negative controls, and each treatment was repeated three times. The inoculated plants were grown in a greenhouse at 24 °C with a 16-h light/8-h dark cycle, and they were observed for 1–2 weeks.

### 4.6. Detection of BrYV in Aphids and Host Plants

After the removal of the aphids, RT-PCR and western blotting were carried out to detect the virus in aphids and host plants. For aphids, virus detection was as described at Section 4.3. For plants, the total RNA was extracted from samples using the TRIzol reagent in accordance with the manufacturer’s protocol. First-strand cDNA was synthesized using 2 μg of DNase treated RNA, oligo d (T) primer or gene-specific primers and M-MLV Reverse Transcriptase as per the protocol. For *Arabidopsis* detection primers were designed as described in Section 4.3 and for Mustard and Chinese cabbage detection primers were designed as BrC2675F:GATTGTTCTGGTTTTGACTGG/BrC3800R: TTATACTCATGGTAGGCCTTGAG. For western blotting assays, total proteins were extracted from systemic leaves of aphid-inoculated *A. thaliana* using 2× sodium dodecyl sulfate (SDS) sample buffer (100 Mm Tris (pH 6.8), 4% (*w*/*v*) SDS, 20% (*v*/*v*) glycerol and 0.2% (*w*/*v*) bromophenol blue) with subsequent heating at 100 °C for 10 min [14,15,16]. Protein separation was conducted in a 12.5% SDS polyacrylamide gel by electrophoresis, and proteins were then transferred to nitrocellulose membrane (GE Healthcare, Buckinghamshire, UK). Western blots were performed with the specific anti-BrYV movement protein (MP) antibody at a 1:1000 dilution as previously described [14], and then incubated with AP-coupled goat anti-rabbit IgG (Sigma-Aldrich, St. Louis, MO, USA) at a 1:3000 dilution. Finally, proteins on the membrane were detected using NBT and BCIP substrates (Sigma-Aldrich).

## 5. Conclusions

BrYV cannot be transmitted mechanically under natural conditions; however, in this research, we developed a simple and reliable approach using the transgenic *Arabidopsis thaliana* harboring the full-length BrYV genome and frozen infected leaves for aphid viral acquisition and transmission. Therefore, this basic research addresses the problem of viral transmission through aphids under natural conditions and provides a long-term viral preservation and utilization technique. This method will aid in accelerating research on interactions among the virus, aphids, and hosts, as well, as the evaluation of host varietal resistance to viruses and aphids.

## Figures and Tables

**Table 1:** Efficiency of the *Agrobacterium tumefaciens*-mediated infiltration of BrYV into inoculated and systemic leaves.

Position	Efficiency (%)
Inoculated leaves (n = 58 plant)	86.34
Systemic leaves (n = 58 plant)	46.75

**Table 2:** Percentages of viruliferous aphids and BrYV-infected plants as a result of aphids feeding on frozen or fresh infected leaves; each experiment was performed three times independently ^1^.

Treatment	Total No. of Aphids	No. of Surviving Aphids *p* < 0.001	Proportion of Viruliferous Aphids (n = 48) (%) *p* = 0.0183	Proportion of Infected Plants (n = 48) (%) *p* = 0.0942
Frozen for 180 d	180 (60 each)	106	87.5	16.6
Frozen for 270 d	180 (60 each)	91	77.08	10.4
Fresh infected leaves	180 (60 each)	166	93.75	33.33
Fresh healthy leaves	180 (60 each)	154	-	-

^1^ Presence of BrYV assessed by RT-PCR.

**Table 3:** Viral transmission frequencies from different numbers of aphids.

Total Number of Insects	No. of Infected Plants (n = 30)	Proportion of Infected Plants (%)
1	9	30
2	24	80
4	27	90
6	30	100
10	30	100

**Table 4:** Minimal inoculation times for viral transmission by six aphids.

Inoculation Time (h)	Efficiency (%) (n = 16)
6	12.5
12	50
24	62.5
48	100

**Table 5:** BrYV transmitted to natural hosts-plants by aphids.

Host Plants	Total Number of Insects	Proportion of Infected Plants (%) n = 36
Mustard	6	94.4
Chinese cabbage	6	100

## Data Availability

The data presented in this study are available within the article.
